# Influence of Arc Power on Keyhole-Induced Porosity in Laser + GMAW Hybrid Welding of Aluminum Alloy: Numerical and Experimental Studies

**DOI:** 10.3390/ma12081328

**Published:** 2019-04-23

**Authors:** Guoxiang Xu, Pengfei Li, Lin Li, Qingxian Hu, Jie Zhu, Xiaoyan Gu, Baoshuai Du

**Affiliations:** 1 School of Materials Science and Engineering, Jiangsu University of Science and Technology, Zhenjiang 212003, China; hfycli@163.com (P.L.); max_linli@163.com (L.L.); huqingxian@126.com (Q.H.); zhujie5858@163.com (J.Z.); liligu1983@163.com (X.G.); 2 State Grid Shandong Electric Power Research Institute, Jinan 250065, China; dubaoshuai@163.com

**Keywords:** hybrid welding, numerical simulation, fluid flow, weld pore, aluminum alloy

## Abstract

A three-dimensional numerical model is used to simulate heat transfer and fluid flow phenomena in fiber laser + gas metal arc welding (GMAW) hybrid welding of an aluminum alloy, which incorporates three-phase coupling and is able to depict the keyhole dynamic behavior and formation process of the keyhole-induced porosity. The temperature profiles and fluid flow fields for different arc powers are calculated and the percent porosities of weld beads were also examined under different conditions by X-ray non-destructive testing (NDT). The results showed that the computed results were in agreement with the experimental data. For hybrid welding, with raising arc power, the keyhole-induced porosity was reduced. Besides the solidification rate of the molten pool, the melt flow was also closely related to weld porosity. A relatively steady anti-clockwise vortex caused by arc forces tended to force the bubble to float upwards at the high temperature region close to the welding heat source, which benefits the escape of the gas bubble from the melt pool. When increasing the arc power, the anti-clockwise region was strengthened and the risk of the gas bubble for capture by the liquid/solid interface underneath the keyhole tip was diminished, which resulted in the lower weld percent porosity.

## 1. Introduction

Welded structures of aluminum alloys are widely used in the automobile, shipbuilding, and aerospace industries among others. Due to high thermal conductivity, the welding heat source with a high power density is more suitable for joining this material. Thus, laser welding is considered one realistic choice for aluminum alloy welding due to its high traveling speed and deep penetration [1,2,3]. However, one of the major problems is the keyhole-induced porosity in weld metal during the deep penetration laser welding of the aluminum alloy, which results from the instability of a laser-induced keyhole. As reported by Ola and Doern [2], this defect is difficult to avoid by the single laser heat source. By coupling a laser beam and an electric arc, the laser + arc hybrid welding process incorporates the advantages of both laser and arc welding processes and overcomes their individual problems [4,5], which has the potential to reduce the porosity of the aluminum alloy weld. Therefore, this obtains increasing attention in the welding of aluminum alloy [6,7,8]. However, compared to welding technology with a single heat source, hybrid welding concerns more process parameters and the optimization of welding parameters is relatively difficult [2,4,6]. Therefore, to improve the stability of the welding quality, it is essential to study the mechanism of arc power on weld porosity in hybrid welding [2].

For hybrid welding of aluminum alloy, some efforts were made to investigate the pore defects. By detecting the keyhole and weld pool behaviors by a micro-focused X-ray transmission real time imaging system, Katayama et al. [8] found that, in the case of high welding current, the gas bubbles easily overflow away due to a large distortion of melt pool free surface during the hybrid laser-arc welding of aluminum alloy. Nevertheless, the resolution of the device needs to be enhanced further and some important details concerning the keyhole oscillation and melt flow pattern cannot be obtained. Ola and Dorn [2] experimentally studied the effects of different parameters on keyhole-induced porosity during hybrid welding of the aluminum alloy and concluded that the role of various welding parameters must be balanced with weld geometry to control porosity during hybrid welding. Similarly, based on the experimental data, Bunaziv et al. [9] claimed that a lower porosity level could be achieved by applying the trailing torch arrangement and the addition of helium to the shielding gas, which has no positive effect in terms of a porosity decrease. Zhang et al. [10] determined the relationship between the molten pool features and weld porosity by means of X-ray non-destructive testing and scanning microscopes with the elemental tracer technique. The researchers demonstrated that the weld porosity is closely related to the shape feature of the molten pool. However, in their study, the dynamic behavior of keyhole was not considered. 

Yet, up to now, the physical phenomenon inside the weld pool is still difficult to observe directly. Although the experimental results can provide the improved understanding of the welding physical process to some extent, they cannot reveal the formation mechanism of porosity in hybrid welding completely. With advancement in computer hard and commercial code, the numerical simulation technology has become one strong tool to investigate the weld formation mechanism [11,12,13,14,15,16,17], which can overcome the shortcomings of experimental methods and show the thermal field and fluid flow pattern in the interior of the weld pool clearly. Nevertheless, due to the complexity of the physical process, few simulation research studies on porosity can be found in the hybrid welding of an aluminum alloy. Xu et al. [18] numerically studied the fluid flow in the weld pool during hybrid welding of aluminum alloy, but their study did not involve porosity. Zhou and Tsai [19] proposed a model to investigate the porosity in deep penetration laser welding and pointed out that porosity would occur when the solidification rate of the molten metal exceeded its backfilling speed. Zhao et al. [20] numerically analyzed the formation process of the bubble in the weld pool for laser welding using a three-dimensional model, which considered the three-phase coupling as well as mass transfer between liquid and vapor phases. They also ascribed the formation of bubble to the sudden shrinkage and collapse of the keyhole. Cho et al. [21] applied an adiabatic bubble model to the simulation of porosity in hybrid welding. However, in their work, the formation process and mechanism of the keyhole-induced porosity was seldom involved and, meanwhile, no comparison between calculated and experimental results of porosity was completed. Similarly, Li et al. [22] developed a three-dimensional model for the formation process of the keyhole-induced porosity in deep penetration laser welding for the T-joint of steel based on Flow-3D software. From the above review, it can be seen that there is still a lack of a mathematical modeling study involving weld defects in hybrid welding of aluminum alloy currently. In this case, it is also worth noting that, due to the insufficiency of thermo-physical properties of material at a high temperature and due to the simplification of the numerical model, the accuracy of simulated results is limited to some degree. In the calculation, the numerical model has to be optimized and modified by comparing predicted results with experimental data. Consequently, the combination of experimental and simulation methods is a better choice to analyze the weld formation mechanism comprehensively.

The main aim of this study is to reveal the influence mechanism of arc power on weld porosity during laser + GMAW hybrid welding of the 6061 aluminum alloy through simulation and experiment methods. For this purpose, a comprehensive three-dimensional heat transfer and fluid flow model is developed and the weld porosity is also examined using X-ray non-detective testing.

## 2. Experimental Methods

### 2.1. Welding Experimentation

**Figure 1:**
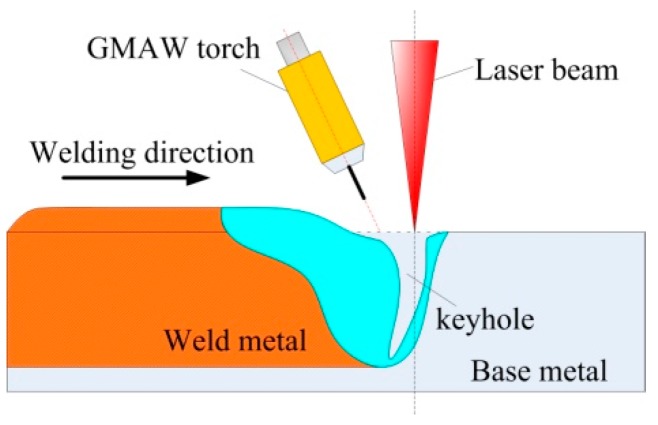
Sketch map of hybrid welding.

Base metal and filler material were 6061 and 5356 aluminum alloys (Aluminum Corporation of China, Beijing, China) respectively. The specimen had dimensions of 150 mm × 40 mm × 6 mm. The diameter of the welding wire was 1.2 mm. A fiber laser beam with a focal spot diameter of 0.3 mm (IPG Photonics Corporation, Oxford, MA, USA) was used in hybrid welding. As seen in Figure 1, laser + GMAW hybrid bead-on-plate welding was performed on the aluminum alloy plate. The laser was in front of the arc and the focal position was set at −1 mm. The angle between the axis of the GMAW torch and the laser beam was 27°. The distance between laser and arc was 2 mm. Pure Ar was used as shielding gas with its flowing rate being 18 L/min. Other welding parameters are listed in Table 1. To guarantee the accuracy of experimental results, for each set of welding parameters, the experiment was repeated three times.

### 2.2. X-Ray Non-Destructive Testing

**Figure 2:**
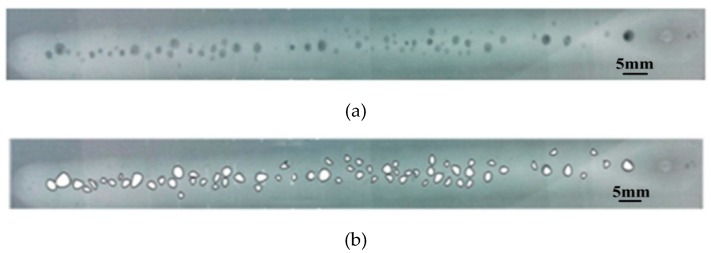
X-ray NDT photos of a typical aluminum alloy weld (**a**) and extraction of the weld pore area (**b**) (*I* = 160 A).

To characterize the porosity in the weldments, the non-destructive testing method was utilized to inspect all the weldments. For each weldment, X-ray film radiography was carried out on the hybrid weld metal using a VJT X-ray inspection system (VJ Technologies Inc., New York City, NY, USA). The X-ray source was operated at 130 kW and 5 mA. Radiographs were produced from the top plane of weldment, which was parallel to the welding direction. Figure 2 gives the X-ray NDT photographic film of a typical aluminum alloy weld. The white area is weld reinforcement and the black spots denote the pore. The length used to calculate the weld percent porosity was 120 mm. According to the standard ISO-10042, the weld percent porosity was defined by the ratio of the pore area to the weld projected area. Figure 2b represents the measured results of the weld pore area. The final weld porosity takes the mean of the measured data for three repeated experiments.

## 3. Mathematical Modeling

It is assumed that both gas and liquid metal are incompressible and Newtonian fluids. The simulations of their velocity fields are conducted according to the laminar flow theory. Meanwhile, the volume of the fluid method is utilized to track the gas-liquid interface.

### 3.1. Governing Equations

The governing equations for energy, momentum, and mass are written as follows [23].

Mass continuity:

(1) $$\frac{\partial \rho }{\partial t}+\frac{\partial \left(\rho u\right)}{\partial x}+\frac{\partial \left(\rho v\right)}{\partial y}+\frac{\partial \left(\rho w\right)}{\partial z}=S_{m}$$

Momentum:

(2) $$\rho \left[\frac{\partial u}{\partial t}+\left(u-u_{0}\right)\frac{\partial u}{\partial x}+v\frac{\partial u}{\partial y}+w\frac{\partial u}{\partial z}\right]=-\frac{\partial p}{\partial x}+\mu \left(\frac{\partial^{2}u}{\partial x^{2}}+\frac{\partial^{2}u}{\partial y^{2}}+\frac{\partial^{2}u}{\partial z^{2}}\right)+S_{x}$$

(3) $$\rho \left[\frac{\partial v}{\partial t}+\left(u-u_{0}\right)\frac{\partial v}{\partial x}+v\frac{\partial v}{\partial y}+w\frac{\partial v}{\partial z}\right]=-\frac{\partial p}{\partial y}+\mu \left(\frac{\partial^{2}v}{\partial x^{2}}+\frac{\partial^{2}v}{\partial y^{2}}+\frac{\partial^{2}v}{\partial z^{2}}\right)+S_{y}$$

(4) $$\rho \left[\frac{\partial w}{\partial t}+\left(u-u_{0}\right)\frac{\partial w}{\partial x}+v\frac{\partial w}{\partial y}+w\frac{\partial w}{\partial z}\right]=-\frac{\partial p}{\partial z}+\mu \left(\frac{\partial^{2}w}{\partial x^{2}}+\frac{\partial^{2}w}{\partial y^{2}}+\frac{\partial^{2}w}{\partial z^{2}}\right)+S_{z}$$

Energy:

(5) $$\rho \left[\frac{\partial H}{\partial t}+\left(u-u_{0}\right)\frac{\partial H}{\partial x}+v\frac{\partial H}{\partial y}+w\frac{\partial H}{\partial z}\right]=\frac{\partial }{\partial x}\left(k\frac{\partial T}{\partial x}\right)+\frac{\partial }{\partial y}\left(k\frac{\partial T}{\partial y}\right)+\frac{\partial }{\partial z}\left(k\frac{\partial T}{\partial z}\right)+S_{V}$$

where *ρ* is the density, *t* is the time, *u*, *v,* and *w* are components of velocity, *p* is the pressure, *μ* is the viscosity, *H* is the enthalpy, *k* is the thermal conductivity, *T* is the temperature, *u*_0_ is the welding speed, *S*_v_ is the heat source terms involving arc and laser heat inputs, *S*_m_ is the mass source, and *S*_x_, *S*_y_, and *S*_z_ are the force source terms, which are expressed below.

(6) $$S_{x}=\left[-\frac{A_{mush}{\left(1-f_{l}\right)}^{2}}{\left(f_{l}^{3}+B\right)}\right]u+F_{x}$$

(7) $$S_{y}=\left[-\frac{A_{mush}{\left(1-f_{l}\right)}^{2}}{\left(f_{l}^{3}+B\right)}\right]v+F_{y}$$

(8) $$S_{z}=\left[-\frac{A_{mush}{\left(1-f_{l}\right)}^{2}}{\left(f_{l}^{3}+B\right)}\right]w+F_{z}+\rho g\beta \left(T-T_{ref}\right)$$

where *A*_mush_ is the mush zone constant, *B* is the positive zero, and *f*_l_ is the fraction of liquid, which is assumed to vary linearly with temperature for simplicity and is explained in Reference [23]. The first terms at the right sides of Equations (6)–(8) is the source terms caused by the frictional dissipation in mushy zone. *F*_x_, *F*_y_, and *F*_z_ are the electro-magnetic force components, which can be available in Reference [24]. The third term at the right side of Equation (8) is the buoyancy force. *T*_ref_ is the reference temperature and *g* is the gravitational acceleration.

Due to the existence of evaporation, the mass transfer between liquid and gas occurs, which is considered through a simple source term in the mass continuity equation used in the work of Zhao et al. [20].

(9) $$S_{m}=\{\begin{array}{c} m_{er}\\ -m_{er} \end{array}$$

where *m*_er_ is the evaporation rate, which takes the negative value in the liquid phase.

### 3.2. Heat Source Model

The double ellipsoid model proposed by Goldak et al. [25] is utilized to depict the arc heat flux distribution, which is given by the equations below.

(10) $$q_{f}=\frac{12\eta_{A}IU}{\pi \left(a_{f}+a_{r}\right)b_{h}c_{h}}\exp\left(-\frac{3x^{2}}{a_{f}^{2}}-\frac{3y^{2}}{b_{h}^{2}}-\frac{3z^{2}}{c_{h}^{2}}\right)x\geq 0$$

(11) $$q_{r}=\frac{12\eta IU}{\pi \left(a_{f}+a_{r}\right)b_{h}c_{h}}\exp\left(-\frac{3x^{2}}{a_{r}^{2}}-\frac{3y^{2}}{b_{h}^{2}}-\frac{3z^{2}}{c_{h}^{2}}\right)x<0$$

where *U*, *I,* and *η* are arc voltage, welding current, and arc efficiency, respectively. Furthermore, *a_f_, a_r_, b_h_,* and *c_h_* are the geometrical parameters of the double ellipsoid.

A cone model with increased peak energy density along its depth direction is selected to represent the laser intensity, which is built by Xu et al. [18] and is given by the equation below.

(12) $$q=Q_{0}\exp\left[-\frac{\ln\left(\chi \right)}{H_{L}}z\right]\exp\left[-\frac{3r^{2}}{{\left(\frac{r_{e}-r_{i}}{H_{L}}z+r_{i}\right)}^{2}}\right]$$

(13) $$Q_{0}=\frac{3Q_{L}\ln\left(\chi \right)}{\pi \left(1-e^{-3}\right)H\left\{r_{e}^{2}-r_{i}^{2}\chi -2\frac{r_{i}-r_{e}}{\ln\left(\chi \right)}\left[r_{e}-r_{i}\chi -\frac{r_{i}-r_{e}}{\ln\left(\chi \right)}\left(1-\chi \right)\right]\right\}}$$

where *Q*_0_ is the constant, *H*_L_ is the heat source height, which is a function of time *t*, *r*_e_ and *r*_i_ are the radii of the heat source top and bottom surfaces, respectively, *χ* is the ratio of maximum heat density at the top surface of the cone to that at its bottom surface, which is set at 1.3 in this study. *Q*_L_ is the effective laser power. 

### 3.3. Boundary Conditions

**Figure 3:**
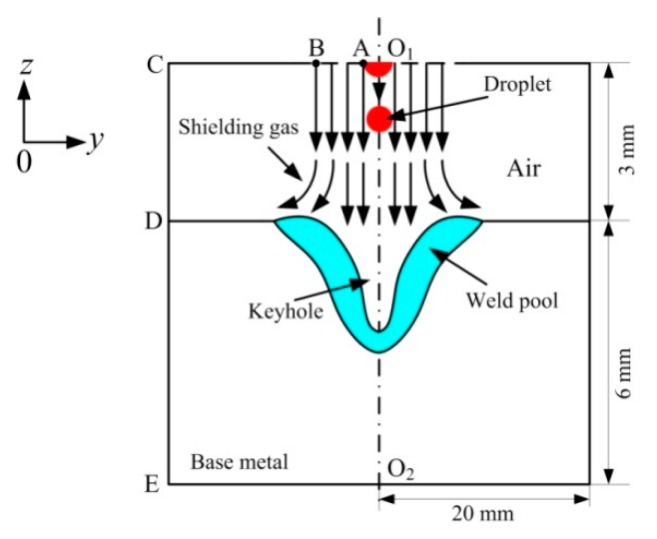
Schematic sketch of the calculation domain.

Figure 3 supplies the schematic sketch of the calculation domain. The domain above the workpiece is filled by air, which is 3 mm in thickness. O_1_A is set as the velocity inlet of the liquid metal with its size being the same as that of the welding wire. BC and CD are set as the pressure outlet. Due to the existence of the symmetrical plane (O_1_O_1_), the width of the calculation domain is set at 20 mm, which is half the specimen width. In simulation, the heat source center is located at the symmetrical plane. Meanwhile, to further enhance the calculation efficiency, the length of the calculation domain takes 50 mm. Because the hybrid weld pool is relatively small at a high welding speed, the size of the calculation domain in this study can guarantee the simulation accuracy of the weld pool dynamic behavior and the formation process of the pore.

#### Weld Pool Free Surface

In this study, arc forces are obtained according to the flow velocity of the arc plasma. Arc plasma is regarded as Ar gas affecting the weld pool free surface at a high speed. O_1_B in Figure 3 is the velocity inlet of gas. According to the previous research of Kim et al. [26], the maximum flow velocity of arc plasma can be calculated by the equation below.

(14) $$v_{mg}=k_{p}I$$

where *k*_p_ is the computational coefficient.

The velocity distribution of arc plasma is described by using a simple formula established by Xu et al [23], which is expressed by the equation below.

(15) $$v_{g}=v_{mg}exp\left(\frac{3r^{2}}{r_{j}^{2}}\right)$$

where *r*_j_ is the distribution radius of the arc plasma. 

Besides arc forces and the droplet impact force, several other forces acting on the free surface of the melt pool are also taken into account in the simulation, including recoil pressure, surface tension, and Marangoni stress.

A simple model is used to deal with the recoil pressure in this study, which is calculated by the following equation adopted in the work of Zhou et al. [19].

(16) $$P_{r}=\frac{A_{0}B_{0}}{\sqrt{T_{w}}}\exp\left(-\frac{U_{0}}{T_{w}}\right)$$

(17) $$U_{0}=\frac{m_{a}H_{v}}{N_{a}k_{b}}$$

where *A*_0_ and *B*_0_ are calculation constants, *U*_0_ is the evaporation constant, *T*_w_ is the temperature of the free surface, *m*_a_ is the mass, *N*_a_ is the Avogadro constant, *k*_b_ is the Boltzmann constant, and *H*_v_ is the vaporization heat. The equations for the other two stresses as well as the energy boundary condition of the weld pool free surface can be available in the study of Xu et al. [18], which are not given here.

## 4. Results and Discussions

The material properties of the aluminum alloy are given in Table 2 and the thermo-physical properties of shielding gas can be determined based on the study of Murphy and Arundell [27].

### 4.1. Experimental Results

**Figure 4:**
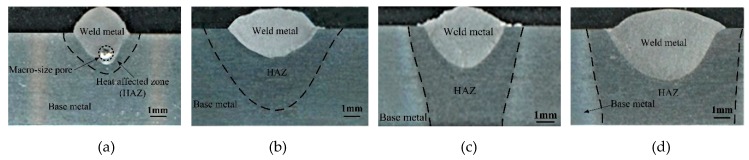
Experimental results of the weld cross-section under different welding currents: (**a**) *I* = 100 A, (**b**) *I* = 140 A, (**c**) *I* = 160 A, and (**d**) *I* = 200 A.

**Figure 5:**
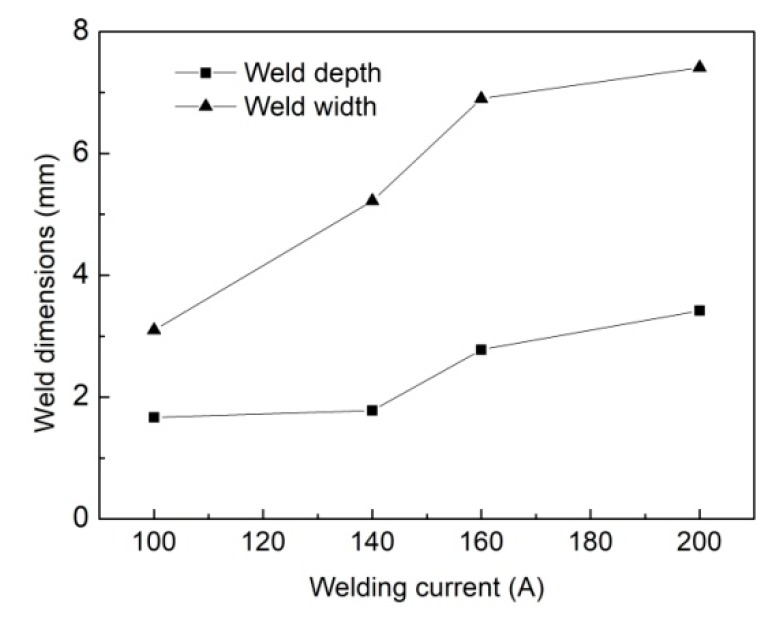
Hybrid weld dimensions for different welding currents.

Figure 4 presents the photographs of weld cross sections under different welding currents and Figure 5 gives the corresponding weld dimensions. The weld size takes the mean of the measured results for three repeated experiments. Both the weld depth and width increase clearly with enhancing arc heat input, especially for the latter. When raising the welding current from 100 A to 200 A, the hybrid weld depth is enhanced by 78% and the weld width grows by 118%. The increased liquid metal volume also suggests more time for the gas bubble to escape from the weld pool.

**Figure 6:**
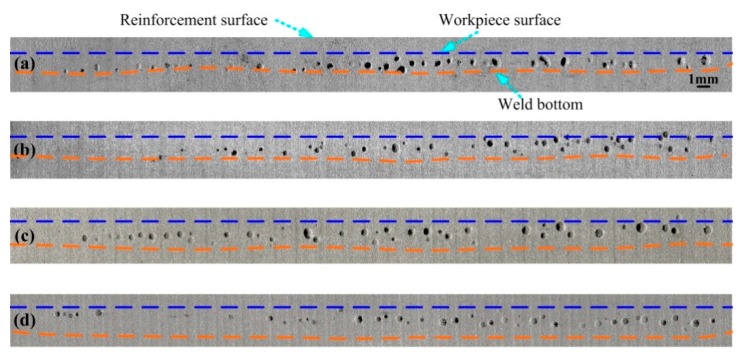
Pore distributions at the longitudinal section of the weld bead under different welding currents: (**a**) *I* = 100 A, (**b**) *I* = 140 A, (**c**) *I* = 160 A, and (**d**) *I* = 200 A.

In addition, Figure 4a shows that the macro-size pores caused by the keyhole are formed in weld metal. This phenomenon can be further confirmed by Figure 6, which shows the macro-photographs of weld longitudinal section. It is observed that, in hybrid welding of the aluminum alloy, there still exists a serious pore defect if the welding parameters do not match well. The pores are mainly distributed at the middle and lower parts of the hybrid weld. As the welding current rises, the porosity tends to be reduced, which will be discussed in the following section.

**Figure 7:**
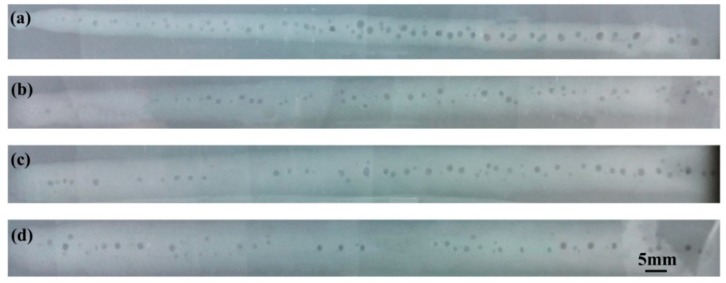
X-ray NDT photos of welds for different welding currents: (**a**) *I* = 100 A, (**b**) *I* = 140 A, (**c**) *I* = 160 A, and (**d**) *I* = 200 A.

To further investigate the influence of arc power on keyhole-induced porosity, the pore size and distribution in the hybrid weld zone were measured using X-ray radiography. Figure 7 shows the X-ray NDT photos of weld beads for different welding currents. It is observed that the pores tend to gather at the region close to the weld center. Meanwhile, corresponding to the observed results shown in Figure 6, the number of pores gradually decreases when increasing the current from 100 A to 200 A. 

**Figure 8:**
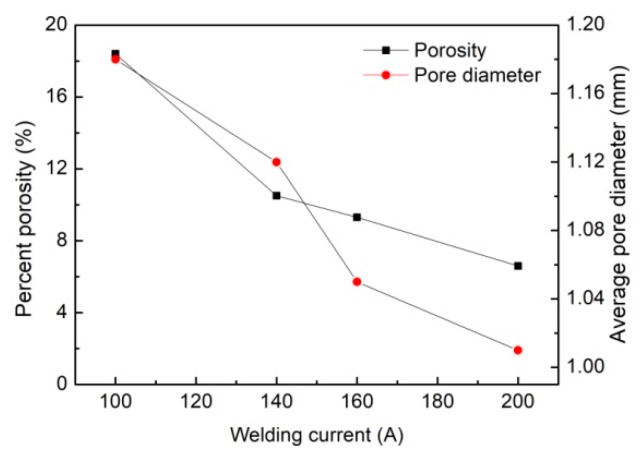
Effect of the welding current on weld percent porosity.

Figure 8 provides the variation trend of the weld percent porosity under different welding currents. It is revealed that the percent porosity in hybrid welding of the aluminum alloy has a sharp decrease from 18.4% to almost 6.6% as the welding current increases from 100 A to 200 A. One commonly accepted reason for this change is that the enhanced heat input from the arc source results in a larger melt pool volume, which increases the time of solidification of the liquid metal pool. This may help the bubbles escape from the molten pool. However, as mentioned above, the fluid flow pattern can also affect the keyhole dynamic behavior and the resulting macro-porosity greatly. Compared with single laser welding, the velocity field and keyhole behavior are more complicated. To comprehend the formation and suppression mechanism of the keyhole fully, the weld pool behavior must be involved in the study. Here, it should be noted that, except for the welding current, the porosity in hybrid welding are related to many other parameters, including laser power, laser-arc distance, the defocusing position, and so on. This study is mainly focused on the impact of arc power on keyhole-induced porosity under the condition that the other parameters are kept at the constants.

### 4.2. Fluid Flow Analysis

**Figure 9:**
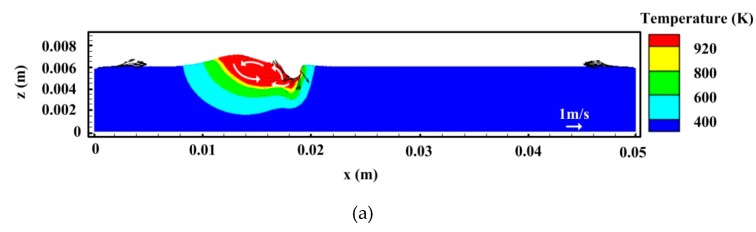

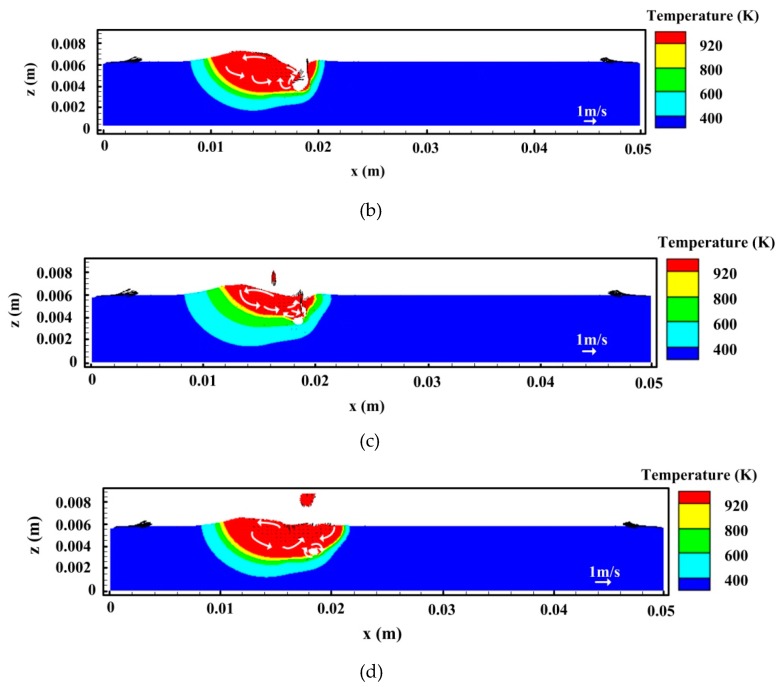
Evolution of the temperature field and fluid flow at the longitudinal section for *I* = 160 A: (**a**) *t* = 0.7 s, (**b**) *t* = 0.71 s, (**c**) *t* = 0.72 s, and (**d**) *t* = 0.82 s.

**Figure 10:**
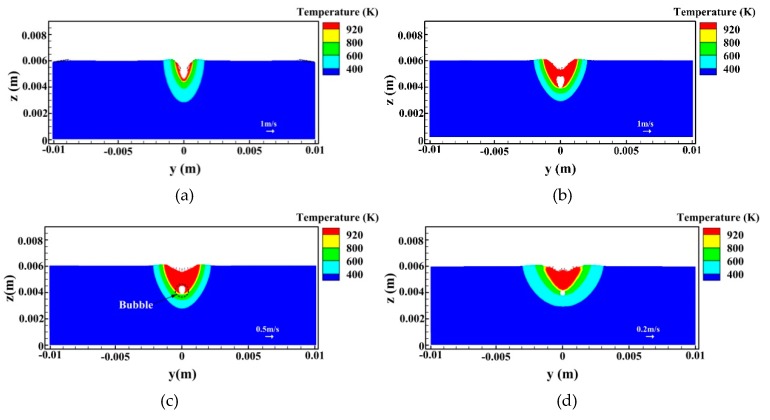
Temperature and velocity fields on the cross section for *I* = 160 A (*x* = 18 mm): (**a**) *t* = 0.7 s, (**b**) *t* = 0.71 s, (**c**) *t* = 0.72 s, and (**d**) *t* = 0.85 s.

Figure 9 and Figure 10 show the temperature and velocity fields in hybrid welding of an aluminum alloy at different times for *I* = 160 A, respectively. As expected, during welding, the keyhole depth is unstable and oscillates within a certain range. This behavior is closely related to the strong instability of the back wall of the keyhole for hybrid welding. Except for the recoil pressure by a laser, there exist several other forces affecting the behavior of the back wall of the keyhole in hybrid welding, including droplet impingement force, arc pressure, electromagnetic force, gravity, and more. It is difficult for these forces to reach the dynamic equivalence, which leads to the complex dynamic behavior of keyhole back all. In addition, due to high welding speed, the lower part of the keyhole is clearly inclined toward the backward, which also reduces the stability of laser density distribution and the resultant recoil pressure in this region to some extent. However, for the front wall of the keyhole, the dynamic behavior is relatively steady and the molten metal always flows downward, which results from the relatively steady laser intensity distributed in the domain. In the case of an inclined keyhole, laser energy is mainly distributed on the front edge of the keyhole due to the action of a direct incoming beam, which results in relatively stability of the forces acting on this region.

In Figure 9, it is observed that, at *t* = 0.7 s, the depth of the keyhole is relatively small. Because of the combined effects of Marangoni stress, shear stress of arc plasma as well as the push of molten metal with high momentum from the front wall, the liquid metal near the back wall of the keyhole moves upwards and then moves backward along the weld pool surface at high velocity. This is similar to the laser beam welding with lower power described by Pang et al. [29]. However, the backward flow of molten metal is strengthened in hybrid welding due to the existence of arc plasma shear stress. Meanwhile, at high welding speed, arc pressure also provides some contributions toward this behavior. Thus, an anti-vortex appears at the middle and rear part. At *t* = 0.71 s, the keyhole depth becomes larger under the action of recoil pressure and the lower part of the keyhole is bent toward the rail of the weld pool, as indicated in Figure 9b. At this moment, the flow pattern of molten metal near the back wall of keyhole changes significantly. For the bent keyhole, the laser beam cannot irradiate the rear wall directly and the heat density is relatively small and unstable at the lower part of the bent keyhole, which reduces the effect of recoil pressure in this region. Consequently, under the action of Marangoni shear stress and surface tension, an upward surface flow appears at the lower part of the rear keyhole wall. Nevertheless, in the upper part of the keyhole, the liquid metal flows downward along the keyhole wall because of the combined effect of recoil pressure, arc pressure, and gravity. Thus, a severe collision of melt flows occurs at the middle part of the rear keyhole wall, producing a hump of molten metal. This behavior tends to cause the collapse of keyhole. Under the action of backward momentum of molten metal from the front keyhole wall, the liquid metal near the keyhole bottom also moves backwards along the weld pool bottom. Meanwhile, Figure 9b also shows that, at the weld pool surface near the keyhole, a strong backward melt flow can be still observed due to the influence of arc plasma shear stress and Marangoni force, which also deflects downwards and backwards at the rail of molten metal pool. In addition, a large anti-vortex still emerges at the middle and back parts of the molten pool. This flow pattern can prevent the bubble from traveling toward the solid/liquid interface at the back of the molten pool to some extent, where most of the bubbles tend to be trapped to form the pores before they escape from the weld pool surface. 

In Figure 9c, it is seen that, at *t* = 0.72 s, the hump of molten metal occurring at the keyhole wall connects the front keyhole wall driven by arc pressure, gravity, and droplet impact forces, which results in an instantaneous closure of the keyhole. Then, the keyhole is divided into two segments, which leads to the formation of the gas bubble near the weld pool bottom. This can be clearly observed in Figure 10c. However, the bubble is unstable at this moment and its behavior and size can be affected greatly by the fluid flow. As stated above, if the bubble fails to escape from the melt pool, it will be trapped as porosity in the weld metal. The forward flow of liquid metal emerging near the weld pool bottom can weaken the tendency of bubble flowing backwards and benefits the escape of bubbles from the liquid metal pool and their merging with a new keyhole to some degree. Nevertheless, due to high welding speed and large thermal conductivity of the aluminum alloy, the solidification rate of the molten pool is still quite large, which leads to the appearance of a relatively severe pore defect in hybrid welding of an aluminum alloy. In addition, in the case of low arc power, the keyhole depth is close to the weld pool depth, which also raises the risk of the gas bubble to be captured by the liquid/solid interface near the keyhole tip, as illustrated in Figure 9c and Figure 10d. From the above analysis, it is seen that, besides a large molten pool volume, the flow pattern in hybrid welding is also a positive factor for the suppression of keyhole-induced porosity.

**Figure 11:**
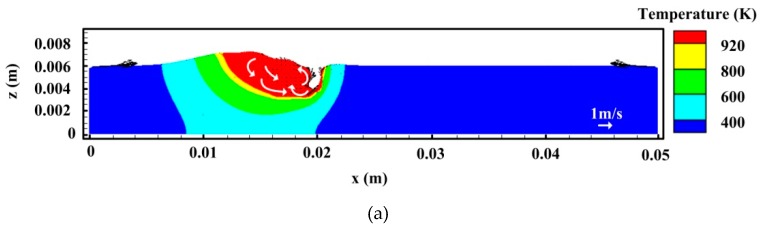

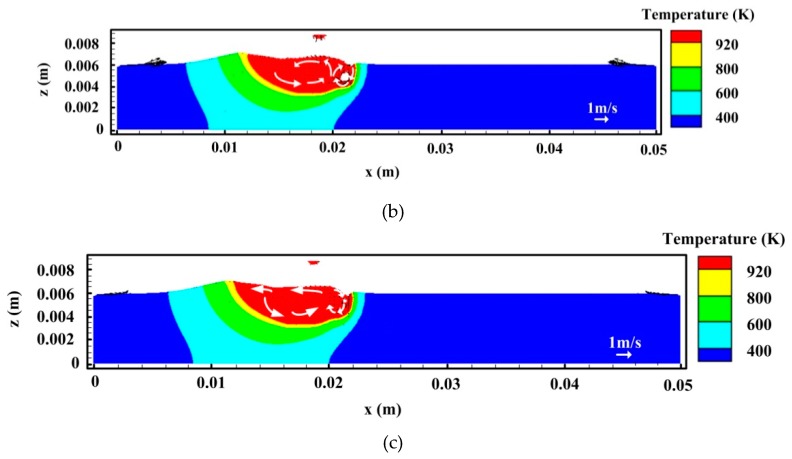
Temperature and velocity fields on the longitudinal section for *I* = 200 A: (**a**) *t* = 0.92 s, (**b**) *t* = 0.93 s, and (**c**) *t* = 0.94 s.

**Figure 12:**
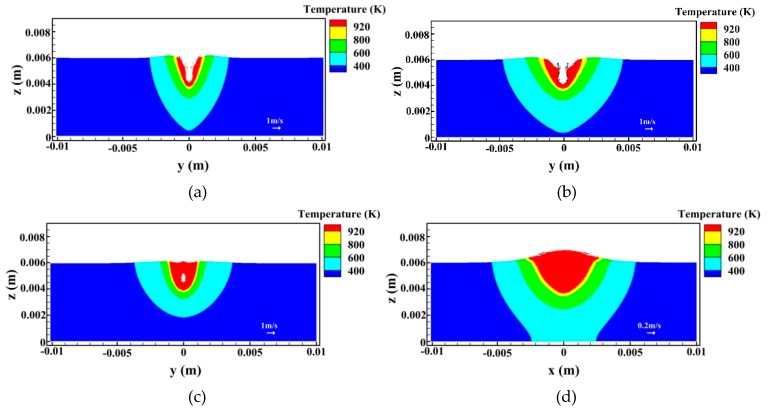
Temperature and velocity fields on the cross section for *I* = 200 A (*x* = 20 mm): (**a**) *t* = 0.92 s, (**b**) *t* = 0.93 s, (**c**) *t* = 0.94 s, and (**d**) *t* = 1.12 s.

Figure 11 and Figure 12 show the evolutions of temperature and velocity fields at longitudinal and cross-sections for *I* = 200 A, respectively. In this case, the feature of the flow field is close to that at 160 A welding current. The keyhole depth still has a fluctuation and the bubble is also generated. Meanwhile, as expected, when the welding current increases to 200 A, the weld pool size has a growth due to enhanced arc heat input, which can provide more time for the bubble to overflow out of the liquid metal pool. As mentioned above, this is usually explained as one important factor responsible for reducing porosity in hybrid welding. However, at a high level of welding current, the arc forces also play a more important role on the fluid flow pattern. At 200 A welding current, the velocity of the backward melt flow tends to be raised further at the weld pool surface due to enhanced arc plasma shear stress and arc pressure. This strengthens the anti-clockwise vortex at the middle and rear parts of the liquid metal to some degree, which leads to a stronger upward flow near the rear keyhole wall, as shown in Figure 11a. As stated above, this behavior helps compel part of the bubbles from the keyhole tip to move toward the weld pool surface in the high temperature region near the hybrid heat source, which reduces the chance of some bubbles to be captured by the solidification surface. Meanwhile, as reported in the work of Pang et al. [29], the relatively strong upward flow of liquid metal at the rear keyhole wall contributes to the enhancement of keyhole stability, which, consequently, has a positive effect on the decrease of porosity. In Figure 11b, it is indicated that a large bubble still appears after the keyhole collapses. Nevertheless, the thickness of liquid metal below the bubble is relatively larger than that at 160 A welding current. Therefore, in this case, the risk of the large bubble to be captured by the liquid/solid interface is further reduced. Due to the influence of complex dynamic flow, the bubble shrinks and will remain as porosity if it is not able to escape from the melt pool.

**Figure 13:**
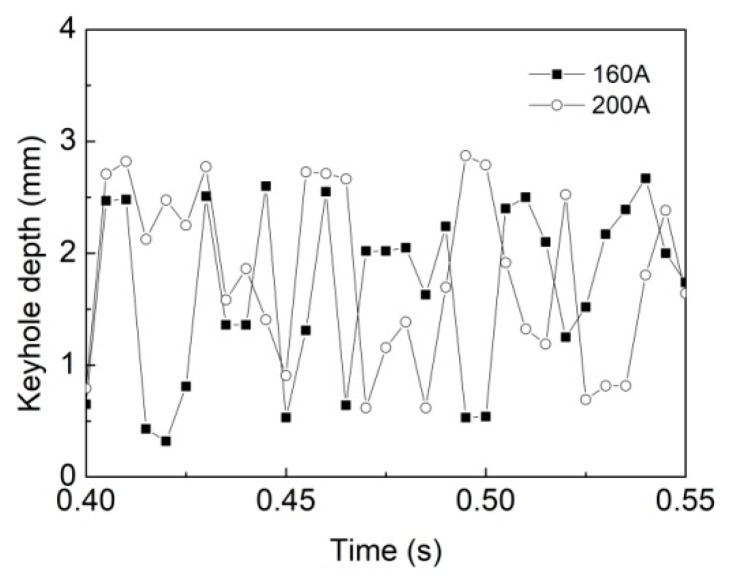
Evolution of the keyhole depth for different welding currents.

Figure 13 gives the evolution of keyhole depth for *I* = 160 A and *I* = 200 A. Compared with that at 160 A welding current, the fluctuation amplitude of keyhole depth changes little at 200 A welding current, but its fluctuation frequency is reduced to some extent, which further validates the increase in keyhole stability. This phenomenon means that, when raising arc power, the number of the gas bubble induced by keyhole collapse can be decreased to some degree, which leads to a lower porosity. However, the bubble and pore sizes have no clear variation. 

### 4.3. Comparisons of Experimental and Simulation Results

**Figure 14:**
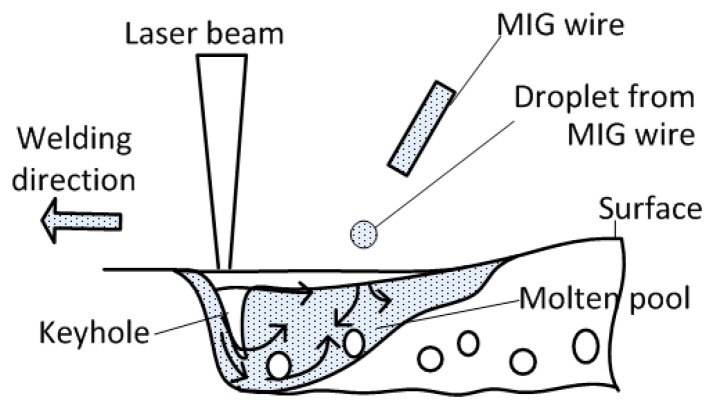
Experimental result of fluid flow for hybrid welding of aluminum alloy [8].

Figure 14 gives the sketch map of a basic fluid flow pattern during hybrid welding of aluminum alloy, which was obtained by Katayama et al. [8] based on the experimentally observed result. It is revealed that the calculated flow pattern in this study is similar to the observation result. Therefore, this validates the accuracy of the above model. 

**Figure 15:**
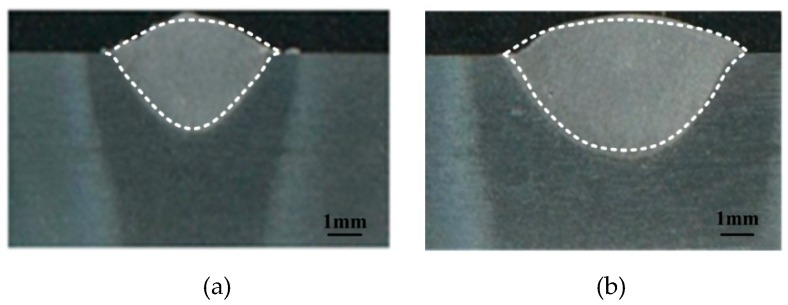
Comparison of the predicted and measured weld profile geometries: (**a**) *I* = 160 A and (**b**) *I* = 200 A.

Meanwhile, to further verify the applicability of the model, the simulated weld shape and size are compared with the measured data. Figure 15 gives a comparison of calculated and experimental weld geometries for varied welding currents and Table 3 supplies the calculated and measured weld cross section sizes. The simulated hybrid weld cross section shapes and dimensions under different welding conditions generally agree well with the experimental data.

## 5. Conclusions

A numerical analysis model is developed to study the transport phenomena in laser + GMAW hybrid welding of aluminum alloy, which considers the multi-phase coupling and can depict the formation process of porosity. An adaptive simplified arc mode is used to describe the shear stress and pressure by arc plasma. The temperature and velocity fields for varied welding currents were calculated. The simulated results were compared with the experimental data to validate the accuracy of the model.

(1) When raising the current from 100 A to 200 A, the hybrid weld pool size increases. Meanwhile, the percent porosity is reduced from 18.4% to 6.6% and the average pore diameter also has a decrease to some degree.

(2) A relatively steady anti-clock vortex appears at the middle and rear part of the hybrid weld pool under the action of arc forces, which tends to hinder the bubble to move toward the domain with low temperature at the back part of the liquid pool. Therefore, this helps the upward floating of the bubble and the resultant reduction of porosity.

(3) At a high level of welding current, besides longer cooling time, the strengthened upward flow of molten metal near the rear keyhole wall and the thicker molten metal layer below the keyhole tip also play a positive role for the decrease of porosity in hybrid welding of aluminum alloy. Meanwhile, with raising arc power, the keyhole stability is increased to some extent.

## Figures and Tables

**Table 1:** Welding parameters used in the experiments.

No.	Welding Current (A)	Laser Power (kW)	Welding Speed (m/min)
**1**	100	2	1.2
**2**	140	2	1.2
**3**	160	2	1.2
**4**	200	2	1.2

**Table 2:** Thermo-physical properties used in the calculation [28].

Name	Value
Density of the aluminum alloy, *ρ*	2700 kg/m^3^
Dynamic viscosity of the liquid phase, *μ*	0.001 kg/(m^3^·s)
Thermal conductivity of the solid phase, *k*_s_	220 W/(m·K)
Thermal conductivity of the liquid phase, *k*_l_	150 W/(m·K)
Specific heat of the liquid phase, c_pl_	1200 J/(kg·K)
Specific heat of the solid phase, *c*_ps_	900 J/(kg·K)
Latent heat of fusion, *L*_m_	3.95 × 10^5^ J/kg
Latent heat of evaporation, *L*_b_	1.07 × 10^7^ J/kg
Thermal expansion, *β*	2.36 × 10^−5^ /K
Solidus temperature, *T*_s_	858 K
Liquidus temperature, *T*_l_	923 K
Ambient temperature, *T*_0_	293 K
Permeability of steel, *μ*_m_	1.26 × 10^−6^ H/m
Surface radiation emissivity, *ε*	0.4
Boltzmann constant, *k_b_*	5.57 × 10^−8^ W/(m^2^·K^4^)
Coefficient in Equation (14), *k*_p_	0.5

**Table 3:** Comparison of the predicted and measured weld cross section sizes.

Welding Current (A)	Weld Penetration Depth (mm)		Weld Width (mm)	
	Measured	Calculated	Measured	Calculated
160	2.4	2.2	6.65	5.9
200	3.2	3.0	7.52	7.23
